# Development and validation of a prognostic nomogram for bone metastasis from lung cancer: A large population-based study

**DOI:** 10.3389/fonc.2022.1005668

**Published:** 2022-09-30

**Authors:** Weihua Li, Zixiang Guo, Zehui Zou, Momen Alswadeh, Heng Wang, Xuqiang Liu, Xiaofeng Li

**Affiliations:** ^1^ Department of Orthopedics, The First Affiliated Hospital of Nanchang University, Nanchang, China; ^2^ Artificial Joints Engineering and Technology Research Center of Jiangxi Province, Nanchang, China; ^3^ Department of Gastroenterology, The First Affiliated Hospital of Nanchang University, Nanchang, China

**Keywords:** lung cancer, SEER database, bone metastasis, prognosis, nomogram

## Abstract

**Background:**

Bone is one of the most common metastatic sites of advanced lung cancer, and the median survival time is significantly shorter than that of patients without metastasis. This study aimed to identify prognostic factors associated with survival and construct a practical nomogram to predict overall survival (OS) in lung cancer patients with bone metastasis (BM).

**Methods:**

We extracted the patients with BM from lung cancer between 2011 and 2015 from the Surveillance, Epidemiology, and End Result (SEER) database. Univariate and multivariate Cox regressions were performed to identify independent prognostic factors for OS. The variables screened by multivariate Cox regression analysis were used to construct the prognostic nomogram. The performance of the nomogram was assessed by receiver operating characteristic (ROC) curve, concordance index (C-index), and calibration curves, and decision curve analysis (DCA) was used to assess its clinical applicability.

**Results:**

A total of 7861 patients were included in this study and were randomly divided into training (n=5505) and validation (n=2356) cohorts using R software in a ratio of 7:3. Cox regression analysis showed that age, sex, race, grade, tumor size, histological type, T stage, N stage, surgery, brain metastasis, liver metastasis, chemotherapy and radiotherapy were independent prognostic factors for OS. The C-index was 0.723 (95% CI: 0.697-0.749) in the training cohorts and 0.738 (95% CI: 0.698-0.778) in the validation cohorts. The AUC of both the training cohorts and the validation cohorts at 3-month (0.842 vs 0.859), 6-month (0.793 vs 0.814), and 1-year (0.776 vs 0.788) showed good predictive performance, and the calibration curves also demonstrated the reliability and stability of the model.

**Conclusions:**

The nomogram associated with the prognosis of BM from lung cancer was a reliable and practical tool, which could provide risk assessment and clinical decision-making for individualized treatment of patients.

## Introduction

Lung cancer ranks second in terms of incidence and first in mortality globally, and most patients are diagnosed at advanced stages due to insidious onset (1). With the advent of the aging era, coupled with environmental deterioration caused by air pollution, tobacco, and lampblack, the incidence of lung cancer is increasing year by year (2, 3). Although non-invasive testing has improved the detection rate of early-stage lung cancer, the 5-year survival rate of lung cancer is only 5%-17% (4, 5). The global cancer burden due to lung cancer is projected to double and top the list by 2050 (6).

Metastasis of lung cancer typically occurs in the bone, liver, respiratory system, nervous system, and adrenal glands, and the incidence of BM in patients with advanced lung cancer is up to 30%-40% (7). Skeletal-related events, such as bone pain, pathological fractures, spinal cord, and nerve root compression, seriously affect the patient’s quality of life (8, 9). Over the past few years, in addition to surgical resection, targeted and checkpoint immunotherapy has provided new treatment ideas for different histological types of lung cancer (10, 11), however, the median survival time for patients with BM is still less than 6 months. Although several studies have explored the incidence and risk factors of BM from lung cancer (12–14), the prognostic factors associated with patient survival have not been extensively studied. Therefore, it is necessary to establish a concise, practical and comprehensive model to predict OS of lung cancer with BM.

Nomograms, as a visual statistical tool, can integrate multiple variables to predict the individual prognosis (15). Compared to conventional staging systems, nomograms facilitate clinical decision-making with increased accuracy and more intuitive prognostic assessment (16, 17). Thus, this study aims to predict the OS of lung cancer patients with BM at 3-month, 6-month and 1-year by constructing a nomogram.

In this study, we obtained the clinical and pathological characteristics of patients with BM from lung cancer diagnosed in 2011-2015 from the SEER database, which contains clinical information on approximately 30% of cancer patients in the United States. Then, we comprehensively analyzed the factors associated with the prognosis of patients with BM from lung cancer and the prognostic nomogram was established to predict the OS of patients.

## Materials and methods

### Patient cohorts

SEER∗Stat software (Version 8.4.0) was applied to extracted data including demographic and clinical characteristics, and the data extraction process was exempt from medical ethics review and did not require informed consent.

**Figure 1:**
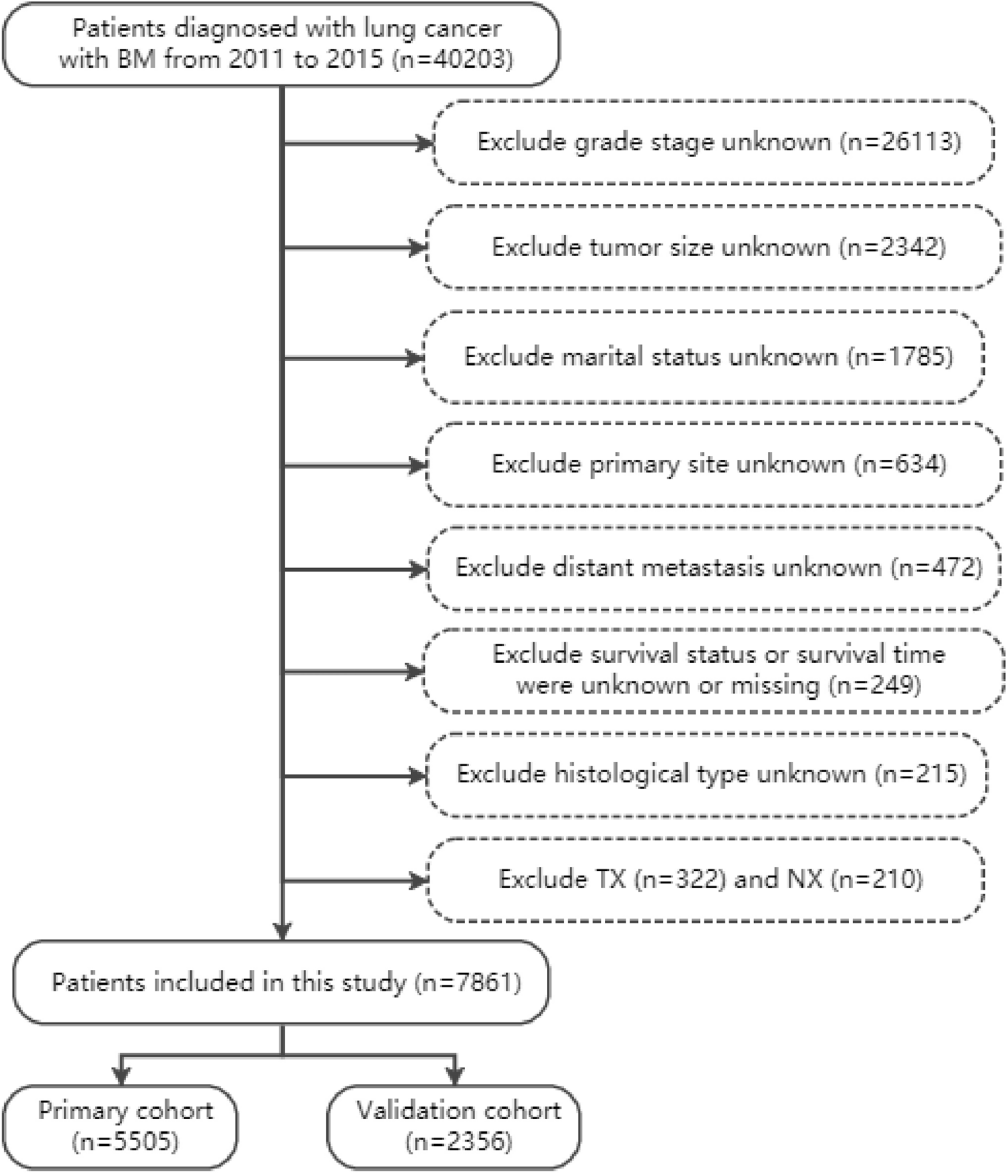
Flowchart for selection of bone metastases from lung cancer.

In accordance with the International Classification of Diseases for Oncology, 3rd Edition, lung cancer was classified as adenocarcinoma, squamous cell carcinoma (SCC), small cell lung cancer (SCLC), large cell lung cancer (LCLC), and non-small cell lung cancer or not otherwise specified lung cancer (NSCLC/NOS). The variables were extracted from the SEER plan, including age, gender, race, marital status, histological type, tumor grade, primary site, laterality, tumor size, TNM stage, distant metastasis, surgery (Code 12, 13, 15, 19-25, 30, 33, 45, 46, 55, 56, 65, 66, 80, 90), radiotherapy and chemotherapy. The following inclusion criteria were applied (1): diagnosed from 2011 to 2015 (2); lung cancer is the only malignant tumor (3); primary diagnosed lung cancer patients with BM. The following should be excluded (1): marital status unknown (n=1785), histological type unknown (n=215), grade stage unknown (n=26113), distant metastasis unknown (n=472), tumor size unknown (n=2342), primary site unknown (n=634) (2); TX (n=322) and NX (n=263) (3); survival status and survival time were unknown or missing (n=249). Finally, 7861 patients with BM from lung cancer were included in our study and were randomly divided into training and validation cohorts in a 7:3 ratio. The specific data extraction and screening are shown in **Figure 1**.

For subsequent analysis, we calculated optimal cutoff values for continuous variables such as age and tumor size using the X-tile software (Yale University, New Haven, USA, version 3.6.1). The optimal cut-off value of age was 70 years (**Supplementary Figures 1A, B**), and the optimal cut-off value of tumor size was 48 mm (**Supplementary Figures 1C, D**).

### Prognostic factors for OS

Univariate and multivariate Cox regression were used to select the prognostic factors for OS. First, we performed univariate Cox regression analysis on all included variables, and the statistically significant variables were further used for multivariate Cox regression analysis. Then, from the results of multivariate Cox regression, variables with P<0.01 were deemed to be independent prognostic factors for OS.

### Nomogram construction

The independent prognostic factors of OS obtained by multivariate Cox regression were used to construct nomogram, and the survival probability of the patients was evaluated according to the total score of each prognostic factor. Then, the model was evaluated by ROC curves, C-index, calibration curves, and DCA.

### Statistical analysis

All statistical analysis processes were completed by packages (rms, ggDCA, survival, survivalROC) in R software version 4.2.0 and Statistical Package for Social Sciences (SPSS, version 26.0). Two-tailed p-value<0.01 was considered statistically significant in this study.

## Results

### Demographic and clinical characteristics

According to inclusion and exclusion criteria, a total of 7861 patients (5505 patients in the training cohort and 2356 patients in the validation cohort) were included in the study. Demographic and clinicopathological characteristics of the training and validation cohorts were shown in **Table 1**, and baselines were comparable between the two cohorts (p>0.01). In this study, the 3-month survival rate of lung cancer patients with BM was 58.6%, the 6-month survival rate was 41.6%, and the 1-year survival rate was 23.6%. The median survival time of patients with BM from lung cancer was 10.3 (95% CI: 9.9-10.6) months, and the vast majority of patients were white (80.8%). More than half of the tumor histological types were adenocarcinomas (54.3%), and the primary sites were mostly located in the upper (60.3%) and lower lobes (30.2%). A large number of patients were grade III (61.3%), T4 (48.8%), or N2 (49.5%), and the rate of simultaneous liver-brain metastasis was less than one-third. In terms of treatment, the proportion of patients without lung surgery was as high as 96.5%, but the proportion of patients with or without radiotherapy was basically the same.

**Table 1:** Demographic and clinicopathological characteristics of the training cohort and validation cohort.

Variable	Total cohort (n = 7861)			Training cohort (n = 5505)			Validation cohort (n = 2356)			X^2^	P-value
**Age**										0.178	0.673
22-70	4536	57.7%		3285	59.7%		1351	57.3%			
71-97	3325	42.3%		2320	42.1%		1005	42.7%			
**Sex**										0.299	0.584
Female	3320	42.2%		2314	42.0%		1006	42.7%			
Male	4541	57.8%		3191	58.0%		1350	57.3%			
**Race**										0.029	0.985
White	6352	80.8%		4451	80.9%		1901	80.7%			
Black	819	10.4%		572	10.4%		274	11.6%			
Other	690	8.8%		482	8.8%		208	8.8%			
**Marital status**										1.293	0.524
Married	4524	57.5%		3169	57.6%		1355	57.5%			
Single	1141	14.5%		813	14.8%		328	13.9%			
SWD	2196	27.9%		1523	27.7%		673	28.6%			
**Grade**										3.590	0.309
I	356	4.5%		241	4.4%		115	4.9%			
II	2012	25.6%		1437	26.1%		575	24.4%			
III	4816	61.3%		3363	61.1%		1453	61.7%			
IV	677	8.6%		464	8.4%		213	9.0%			
**Laterality**										2.333	0.127
Left	3351	42.6%		2316	42.1%		1035	43.9%			
Right	4510	57.4%		3189	57.9%		1321	56.1%			
**Primary site**										0.584	0.900
Main bronchus	386	4.9%		275	5.0%		111	4.7%			
Upper lobe	4739	60.3%		3307	60.1%		1432	60.8%			
Middle lobe	362	4.6%		252	4.6%		110	4.7%			
Lower lobe	2374	30.2%		1671	30.4%		703	29.8%			
**Tumor size**										2.151	0.143
≤48mm	4018	51.1%		2784	50.6%		1234	52.4%			
>48mm	3843	48.9%		2721	49.4%		1122	47.6%			
**Histologic type**										1.247	0.870
Adenocarcinoma	4272	54.3%		2991	54.3%		1281	54.4%			
SCC	1583	20.1%		1101	20.0%		482	20.5%			
SCLC	730	9.3%		505	9.2%		225	9.6%			
LCLC	159	2.0%		112	2.0%		47	2.0%			
NSCLC/NOS	1117	14.2%		796	14.5%		321	13.6%			
**Stage T**										2.658	0.447
T1	865	11.0%		603	11.0%		262	11.1%			
T2	2603	33.1%		1797	32.6%		806	34.2%			
T3	560	7.1%		403	7.3%		157	6.7%			
T4	3833	48.8%		2702	49.1%		1131	48.0%			
**Stage N**										0.362	0.948
N0	1569	20.0%		1107	20.1%		462	19.6%			
N1	730	9.3%		514	9.3%		216	9.2%			
N2	3893	49.5%		2719	49.4%		1174	49.8%			
N3	1669	21.2%		1165	21.2%		504	21.4%			
**Surgery**										4.840	0.028
No	7586	96.5%		5296	96.2%		2290	97.2%			
Yes	275	3.5%		209	3.8%		66	2.8%			
**Brain metastasis**										1.188	0.276
No	6118	77.8%		4266	77.5%		1852	78.6%			
Yes	1743	22.2%		1239	22.5%		504	21.4%			
**Liver metastasis**										0.814	0.367
No	5892	75.0%		4142	75.2%		1750	74.3%			
Yes	1969	25.0%		1363	24.8%		656	27.8%			
**Chemotherapy**										0.867	0.352
No/unknown	3128	39.8%		2172	39.5%		956	40.6%			
Yes	4733	60.2%		3333	60.5%		1400	59.4%			
**Radiotherapy**										0.216	0.642
No/unknown	3695	47.0%		2597	47.2%		1098	46.6%			
Yes	4166	53.0%		2908	52.8%		1258	53.4%			

SDW, separated, divorced, or widowed; SCC, squamous cell carcinoma; SCLC, small cell lung cancer; LC, large cell; NSCLC/NOS, non-small cell lung cancer/not otherwise specified.

### Identifying independent prognostic factors

Independent prognostic factors in lung cancer patients with BM were analyzed by univariate and multivariate Cox proportional hazard regression (**Table 2**). And the results demonstrated that age, sex, race, grade, tumor size, histological type, T stage, N stage, surgery, brain metastasis, liver metastasis, chemotherapy and radiotherapy were independent prognostic factors for OS, and the visualization results were displayed in the forest plot (**Supplementary Figure 2**).

**Table 2:** Univariate and multivariate Cox proportional hazards regression analysis of prognosis of lung cancer with bone metastasis in the training cohort.

Variable		Univariate analysis				Multivariate analysis		
		HR	95% CI	P-value		HR	95% CI	P-value
**Age**								
22-70		Ref				Ref		
71-97		1.403	1.328- 1.481	<0.001*		1.238	1.169- 1.311	<0.001*
**Sex**								
Female		Ref				Ref		
Male		1.234	1.168- 1.303	<0.001*		1.259	1.190- 1.333	<0.001*
**Race**								
White		Ref				Ref		
Black		1.017	0.931-1.110	0.717		0.919	0.840- 1.005	0.065
Other		0.652	0.591- 0.720	<0.001*		0.680	0.616- 0.751	<0.001*
**Marital status**								
Married		Ref				Ref		
Single		1.125	1.040- 1.217	<0.01*		1.101	1.016- 1.1936	0.019
SWD		1.195	1.123- 1.271	<0.001*		1.071	1.004- 1.1428	0.036
**Grade**								
I		Ref				Ref		
II		1.094	0.937- 1.239	0.297		1.006	0.874- 1.158	0.934
III		1.364	1.193- 1.561	<0.001*		1.231	1.074- 1.410	0.003*
IV		1.511	1.288- 1.771	<0.001*		1.377	1.150- 1.649	<0.001*
**Laterality**								
Left		Ref				–	–	–
Right		0.997	0.944- 1.052	0.906		–	–	–
**Primary site**								
Main bronchus		Ref				–	–	–
Upper lobe		0.858	0.757- 0.972	0.016		–	–	–
Middle lobe		0.834	0.700- 0.992	0.040		–	–	–
Lower lobe		0.873	0.767- 0.994	0.040		–	–	–
**Tumor size**								
≤48mm		Ref				Ref		
>48mm		1.318	1.249- 1.391	<0.001*		1.186	1.119- 1.257	<0.001*
**Histologic type**								
Adenocarcinoma		Ref				Ref		
SCC		1.510	1.407- 1.620	<0.001*		1.250	1.161- 1.345	<0.001*
SCLC		1.285	1.168- 1.413	<0.001*		1.051	0.929- 1.188	0.434
LCLC		1.250	1.032- 1.514	0.022		1.022	0.837- 1.248	0.831
NSCLC/NOS		1.391	1.284- 1.506	<0.001*		1.186	1.093- 1.288	<0.001*
**Stage T**								
T1		Ref				Ref		
T2		1.312	1.193- 1.442	<0.001*		1.098	0.994- 1.213	0.066
T3		1.383	1.215- 1.574	<0.001*		1.175	1.025- 1.347	0.021
T4		1.431	1.306- 1.567	<0.001*		1.274	1.156- 1.404	<0.001*
**Stage N**								
N0		Ref				Ref		
N1		1.008	0.905- 1.122	0.889		1.026	0.921- 1.143	0.647
N2		1.243	1.157- 1.335	<0.001*		1.257	1.167- 1.354	<0.001*
N3		1.178	1.083- 1.281	<0.001*		1.259	1.153- 1.375	<0.001*
**Surgery**								
No		Ref				Ref		
Yes		0.534	0.460- 0.620	<0.001*		0.533	0.458- 0.621	<0.001*
**Brain metastasis**								
No		Ref				Ref		
Yes		1.098	1.03- 1.171	0.004*		1.263	1.178- 1.354	<0.001*
**Liver metastasis**								
No		Ref				Ref		
Yes		1.522	1.429- 1.62	<0.001*		1.516	1.420- 1.619	<0.001*
**Chemotherapy**								
No/unknown		Ref				Ref		
Yes		0.363	0.343- 0.384	<0.001*		0.333	0.314- 0.354	<0.001*
**Radiotherapy**								
No/unknown		Ref				Ref		
Yes		0.905	0.857- 0.955	<0.001*		0.917	0.866- 0.971	0.003 *

SDW, separated, divorced, or widowed; SCC, squamous cell carcinoma; SCLC, small cell lung cancer; LC, large cell; NSCLC/NOS, non-small cell lung cancer/not otherwise specified;*: statistical difference.

### Construction and evaluation of nomogram

**Figure 2:**
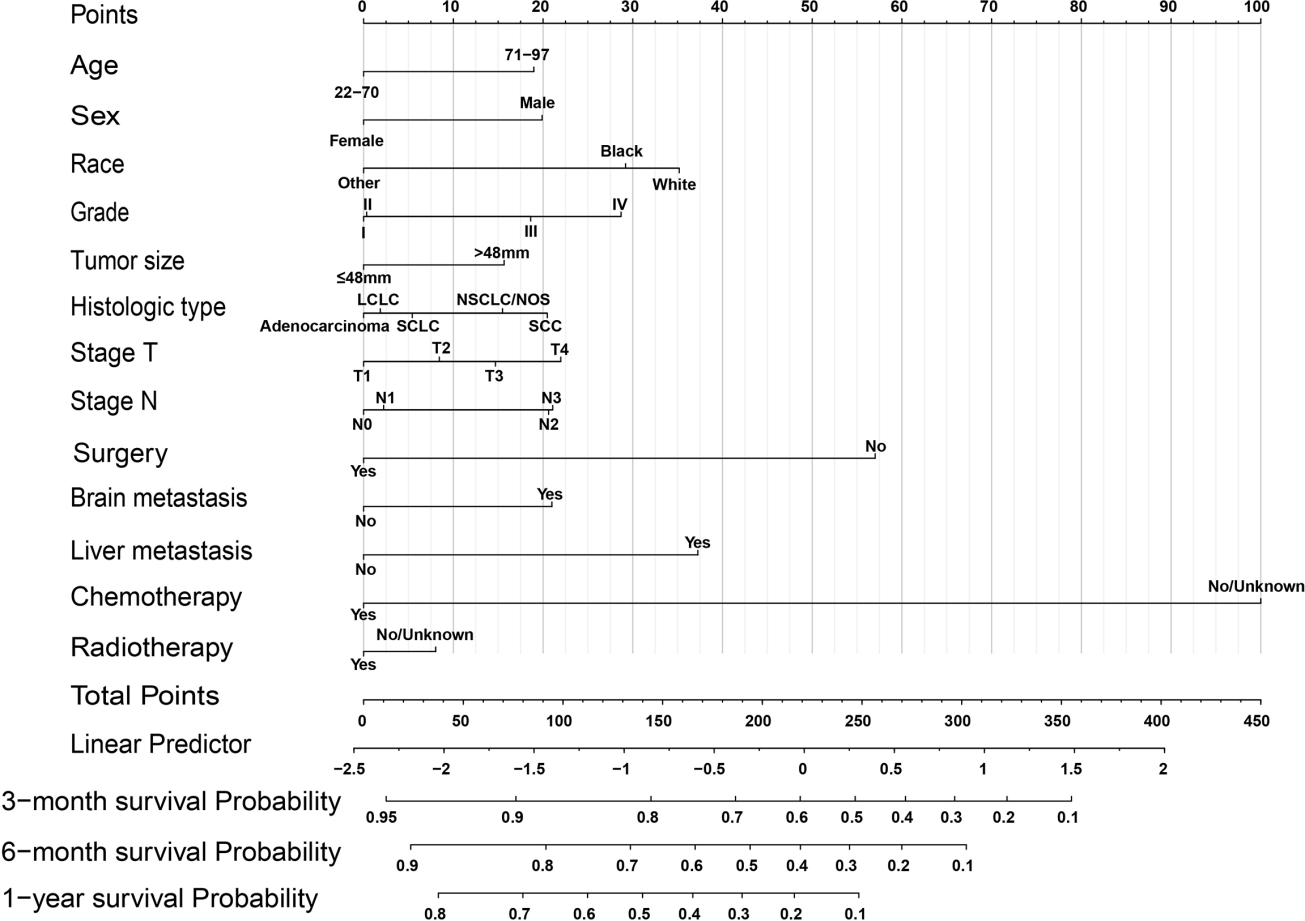
OS nomogram for lung cancer with bone metastasis. SCC, squamous cell carcinoma; SCLC, small cell lung cancer; LC, large cell; NSCLC/NOS, non-small cell lung cancer/not otherwise specified.

Significant prognostic factors from the multivariate Cox regression were acquired to construct a comprehensive nomogram (**Figure 2**). In the predictive model, we predicted the patients’ OS by calculating the total score of each variable, and we found that surgery, liver metastasis, and chemotherapy had great predictive value. **Table 3** shows the specific scores for each variable and the survival probability corresponding to the total score.

**Table 3:** Nomogram scoring system.

Variable	Points		Variable	Points		Variable	Points
**Age**			**Sex**			**Tumor size**	
22-70	0		Female	0		≤48mm	0
71-97	19		Male	19.9		>48mm	15.7
**Race**			**Histologic type**			**Histologic type**	
White	35.2		Adenocarcinoma	0		LCLC	1.9
Black	29.2		SCC	20.5		NSCLC/NOS	15.5
Other	0		SCLC	5.42			
**Grade**			**Stage T**			**Stage N**	
I	0		T1	0		N0	0
II	0.4		T2	8.4		N1	2.2
III	18.6		T3	14.7		N2	20.6
IV	28.7		T4	22.0		N3	21.1
**Surgery**			**Brain metastasis**			**Liver metastasis**	
No	57		No	0		No	0
Yes	0		Yes	21		Yes	37.3
**Chemotherapy**			**Radiotherapy**				
No/unknown	100		No/unknown	8			
Yes	0		Yes	0			
**3-month Survival Probability**			**6-month Survival Probability**			**1-year Survival Probability**	
0.10	355		0.10	302.3		0.10	248.4
0.20	322.7		0.20	270.0		0.20	216.0
0.30	296.5		0.30	243.7		0.30	189.8
0.40	271.8		0.40	219.1		0.40	165.1
0.50	246.6		0.50	193.9		0.50	139.9
0.60	219.0		0.60	166.3		0.60	112.3
0.70	186.5		0.70	133.8		0.70	79.9
0.80	144.2		0.80	91.4		0.80	37.5
0.90	76.4		0.90	23.6			
0.95	11.3						

SCC, squamous cell carcinoma; SCLC, small cell lung cancer; LC, large cell; NSCLC/NOS, non-small cell lung cancer/not otherwise specified.

**Figure 3:**
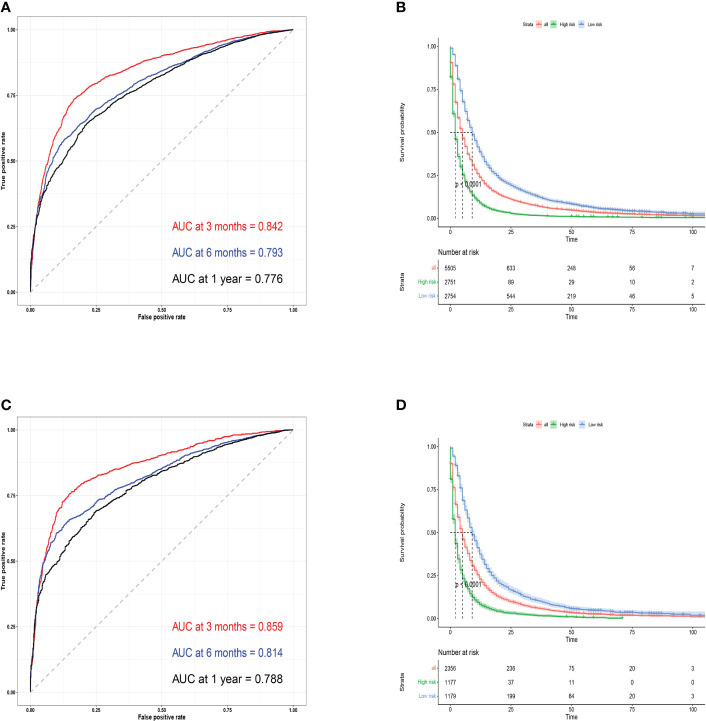
Training cohort ROC curves **(A)** and validation cohort ROC curves **(C)** for predicting 3- month, 6-month, and 1-year OS. Kaplan-Meier survival curves for training **(B)** and validation cohorts **(D)**.

Next, to test the predictive performance of the nomogram, AUC, C-index, and calibration curves were performed. And the C-index was 0.723 (95%CI: 0.697-0.749) in the training cohort and 0.738 (95%CI: 0.698-0.778) in the validation cohort. The AUC values for 3-month, 6-month, and 1-year were 0.842, 0.793, and 0.776 in the training cohort and 0.859, 0.814, and 0.788 in the validation cohort, which indicated that the nomogram had a good predictive ability in OS for patients with BM from lung cancer (**Figures 3A, C**). Furthermore, the 3-month, 6-month, and 1-year calibration curves also demonstrated the reliability of the nomogram (**Supplementary Figures 3A-F**). At different thresholds, the decision curves were all located above the None line and the All line, indicating that the model had certain clinical applicability (**Supplementarys Figure 4A-F**).

Finally, we categorized the training cohort and the validation cohort into high-risk and low-risk groups according to the median survival time using R software, and the high-risk patients had a worse prognosis than the low-risk patients (P<0.001). The predicted probability of OS within one year had a significant downward trend (**Figures 3B, D** **)**, which also indicates that the model had an effective predictive ability for patients with BM from lung cancer.

### Survival analysis

To further verify the relationship between the variables included in the nomogram and survival, we stratified the variables and performed the K-M survival analysis. The results showed that age, sex, race, grade, tumor size, histologic type, TN stage, distant metastasis, and treatment method were all significantly correlated with the survival of patients with BM from lung cancer (P<0.001) (**Supplementary Figures 5A-M**). At the same time, we also found that these patients had a higher probability of survival, characterized by younger age, female gender, other races, smaller tumor size, lower pathological stage and grade, adenocarcinoma, no liver or brain metastases, and treatment with surgery and chemoradiotherapy. In the survival curves, patients who underwent surgery, chemotherapy and no liver metastases had a lower risk of death (**Supplementary Figures 5I, K, L**), but some of the curves partially cross, which means there may be multiple interference factors.

## Discussion

Advanced lung cancer patients die mostly from distant metastases, and their median survival time is only five months (18). Bone is one of the common sites of metastasis from advanced lung cancer, statistics showed that more than a third of lung cancer patients died early due to BM (8, 19).

In recent years, erlotinib, gefitinib and afatinib, three novel epidermal growth factor receptor (EGFR) tyrosine kinase inhibitors (TKIs), have shown good efficacy in patients with distant metastasis, and progression-free survival and OS were longer after treatment (20, 21). In two randomized controlled clinical trials, bisphosphonates also can reduce pain and inflammation, improve blood calcium levels and immunity, and greatly improve the quality of life of lung cancer patients with BM (22, 23). In addition, high‐resolution chest CT, whole-body bone scan, and serological molecular model provide convenience for the early diagnosis of distant metastasis of lung cancer (24, 25). However, the long-term survival rate and prognosis of patients are still unsatisfactory. Therefore, it is imperative to determine the prognostic factors of patients with BM from lung cancer. Here, we constructed an easy-to-use nomogram to predict the prognosis of patients, hoping to provide a reference for clinicians to formulate individualized treatment plans.

In this study, age, sex, race, grade, tumor size, histologic type, T stage, N stage, surgery, brain metastasis, liver metastasis, chemotherapy, and radiotherapy were closely associated with the OS of patients with BM from lung cancer. Among these, age over 70 years, male, poor differentiation grade, liver metastasis, brain metastasis, no surgery and no chemoradiotherapy could significantly reduce the survival rate of patients. Furthermore, higher scores for surgery, liver metastasis, and chemotherapy mean that these variables have greater prognostic value, which was consistent with the findings of Shen H et al. on prognostic factors of brain metastasis from lung cancer (26).

According to most previous literature reports, lung adenocarcinoma has the highest risk of bone metastasis, but its survival rate is better than other types of lung cancer (27–29). However, the study of Qiu Dong showed that the survival rate of patients with BM from lung adenocarcinoma was lower than that of patients with other types of NSCLC, which was contrary to the conclusions of most previous literature (30). From our results, we found that SCC had the worst prognosis, adenocarcinoma had the best prognosis, and SCLC was in between. Additionally, liver is an important immune monitoring organ in the human body. Once liver metastases occur, the OS of patients with BM from lung cancer will be significantly shortened even if no metastases occur in other parts (31, 32). According to our study, more than three-quarters of patients with BM had concurrent liver or brain metastases, which may be the reason for the higher scores for liver metastasis in our model.

Patients with distant metastases are generally considered inoperable, however, surgery, radiotherapy, and chemotherapy as protective factors can significantly improve the overall survival rate of patients with BM (33). A recent propensity score-matched study showed that lobectomy/bilobectomy with regional lymph node resection significantly improved the prognosis of lung cancer patients with BM (34), and only 2.2% of the population underwent primary site surgery, which was comparable to the 3.5% in our study. However, in a study of radiotherapy combined with immunotherapy for advanced NSCLC, the objective response rate of brain tissue was 48.65%, while that of bone tissue was only 17.07%, which may indicate that BM was not sensitive to radiotherapy (35). But previous studies suggested that single or short-term palliative radiotherapy was beneficial in patients with advanced cancer, which may require further prospective studies (36, 37). At present, most scholars still use chemotherapy alone or sequential chemoradiotherapy as the best treatment strategy for patients with advanced lung cancer.

In the nomogram, we predicted 3-month, 6-month, and 1-year OS in lung cancer patients with BM. However, Si Shi et al. developed a nomogram to predict the 1-year, 3-year, and 5-year OS of NSCLC with BM, and only 484 patients were enrolled (38). As far as we know, the median survival time of most patients was less than 6 months, the prediction of 3-year and 5-year OS cannot cover the vast majority of patients with BM. Previous studies had mainly focused on the analysis of risk factors and prognostic factors for BM in a specific histological type of lung cancer. Although narrowing the study object can improve the accuracy of the model, this may limit the included data and population to a certain extent, which will undoubtedly lead to a reduction in the scope of applicability of the model. We hope to cover as many people as possible to achieve the best model applicability.

In order to further validate the validity of the model, in addition to the ROC curve, we also introduced the C-index, calibration curve and DCA. In both the training cohort and the validation cohort, the C-index is greater than 0.7 (0.723 vs 0.738). The calibration curves of 3-month, 6-month, and 1-year were basically parallel to the 45-degree dotted line in the figure, which showed that the model construction had high accuracy and stability. As an advanced tool to detect whether the model is effective in clinical decision-making, DCA can better reflect the clinical practicality of the nomogram (39). The basic interpretation of the DCA is that given a chosen risk threshold, the curve displays the net benefit of using the risk model with that risk threshold, and the ideal predictive model has a higher net benefit than both extremes(”intervention for all” and “intervention for none”)at different threshold probabilities (40, 41). Our results showed that the nomogram has excellent net benefit at different threshold probabilities in both the training and validation cohorts, implying that the nomogram predicted survival with better clinical benefit.

Although we used a large sample of data from the SEER database, and performed effective analysis and internal validation of the model, some limitations inevitably existed in this study. First, this study was a retrospective study, and the process of data extraction and screening may be subject to selection bias due to the different research directions of doctors. Second, the patient’s treatment method only included whether to perform surgery, radiotherapy or chemotherapy. The specific type of surgery, radiotherapy dose, and chemotherapy drugs were not mentioned, and emerging targeted therapy and immunotherapy were not involved. Third, biochemical indicators such as alkaline phosphatase, lactate dehydrogenase, and tumor markers were closely related to the prognosis of patients with BM, but these biochemical tests were not included in the database, and records of asymptomatic patients with BM were lacking. Fourth, the lack of information such as comorbidities and gene mutations also limited the selection and analysis of data in this study to a certain extent. Fifth, the patient population in the database was mainly from the United States, and the applicability of the model to other ethnicities and regions needs further prospective clinical trials for external validation analysis.

## Conclusions

In conclusion, we developed and validated a simple-to-use nomogram for predicting the OS of patients with BM from lung cancer. The nomogram is more comprehensive with higher accuracy, which could provide clinical decision-making for individualized treatment of patients.

## Data availability statement

Publicly available datasets were analyzed in this study. This data can be found here: https://seer.cancer.gov/data/.

## Author contributions

WL and ZG collected and analyzed the data, and drafted the manuscript. ZZ, MA, and HW interpreted the results, handled the picture, and checked the data. XQL and XFL conceived and designed the study, and revised the manuscript. All authors contributed to the article and approved the submitted version.

## Funding

This work was supported by the National Natural Science Foundation of China [Grant Number: 82160173]; the Natural Science Foundation of Jiangxi Province, [Grant Number: 20212BAB216019, 20171BAB205033].

## Conflict of interest

The reviewer CY declared a shared parent affiliation with all authors to the handling editor at the time of review.

The remaining authors declare that the research was conducted in the absence of any commercial or financial relationships that could be construed as a potential conflict of interest.

## Publisher’s note

All claims expressed in this article are solely those of the authors and do not necessarily represent those of their affiliated organizations, or those of the publisher, the editors and the reviewers. Any product that may be evaluated in this article, or claim that may be made by its manufacturer, is not guaranteed or endorsed by the publisher.
