# Supplementary material for: Development and validation of a prognostic nomogram for bone metastasis from lung cancer: A large population-based study
Source: Front Oncol. 2022 Sep 30;12:1005668. doi: 10.3389/fonc.2022.1005668 (PMC9561801; doi:10.3389/fonc.2022.1005668)
Supplement: Supplementary file 1 [file DataSheet_1.docx]

Supplementary Material

## Supplementary Figures

**Supplementary Figure 1** Estimation of the appropriate cut-off value for the age and tumor size by using X-tile analysis. The optimal cut-off value of age was 70 years (A, B). The optimal cut-off value of tumor size was 48 mm (C, D).


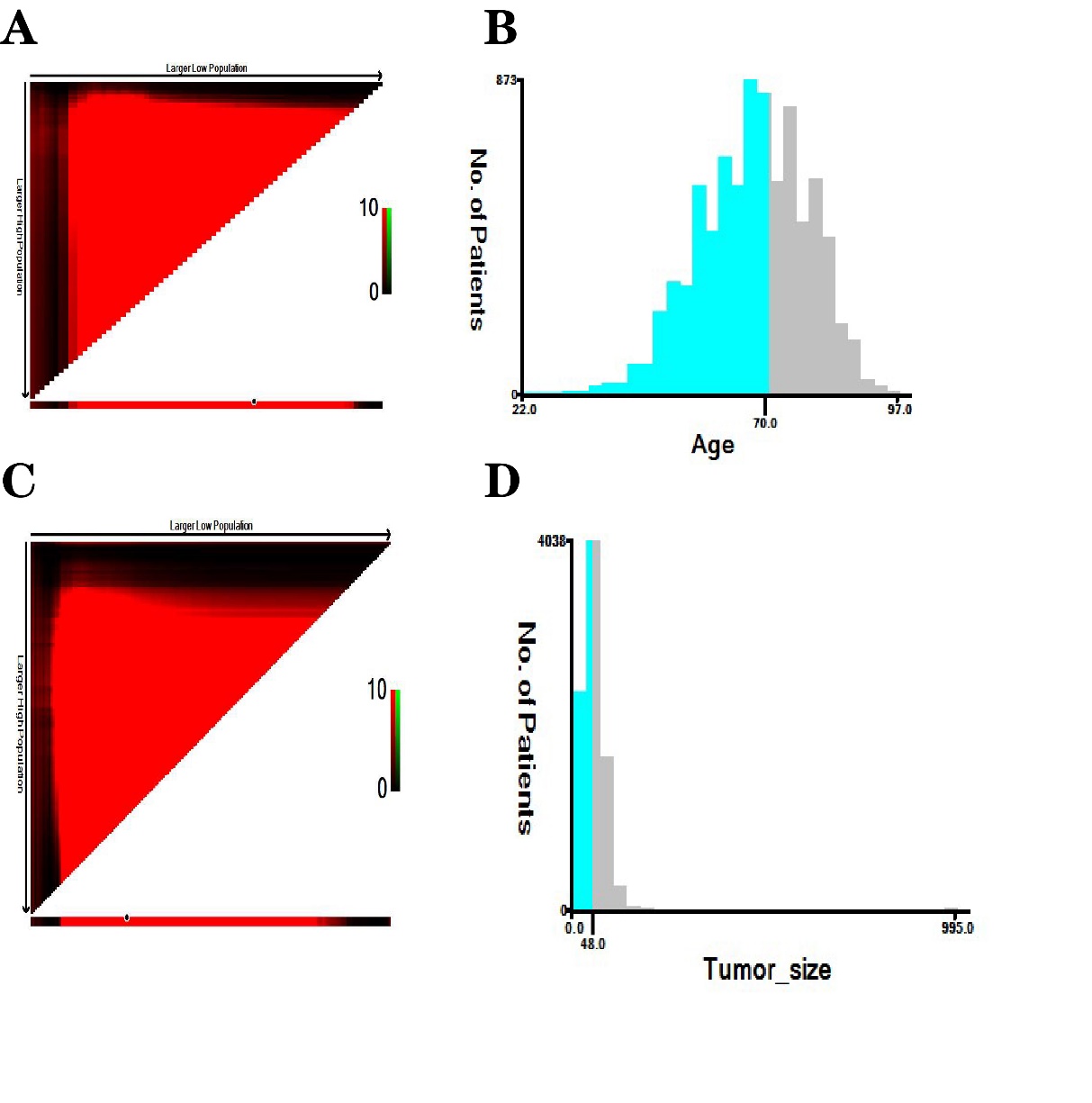


**Supplementary Figure 2** Forest plot depicting the effects of different prognosis factors. SCC: squamous cell carcinoma; SCLC: small cell lung cancer; LC: large cell; NSCLC/ NOS: non-small cell lung cancer/not otherwise specified；*: statistical difference.


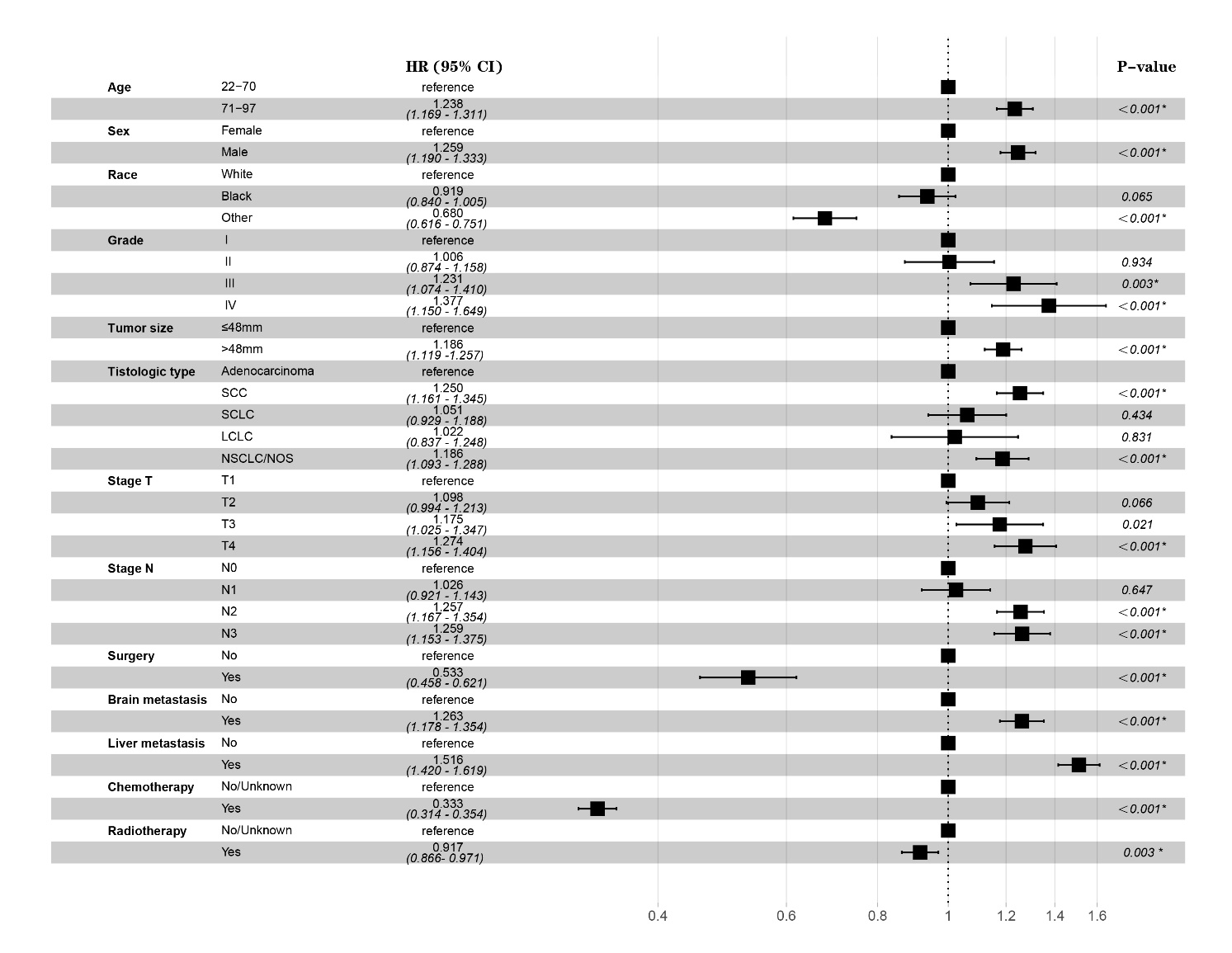


**Supplementary Figure 3** Calibration curves. The calibration curves of the nomogram for 3-month, 6-month, and 1-year OS prediction of the training cohort (A-C), validation cohort (D-F).


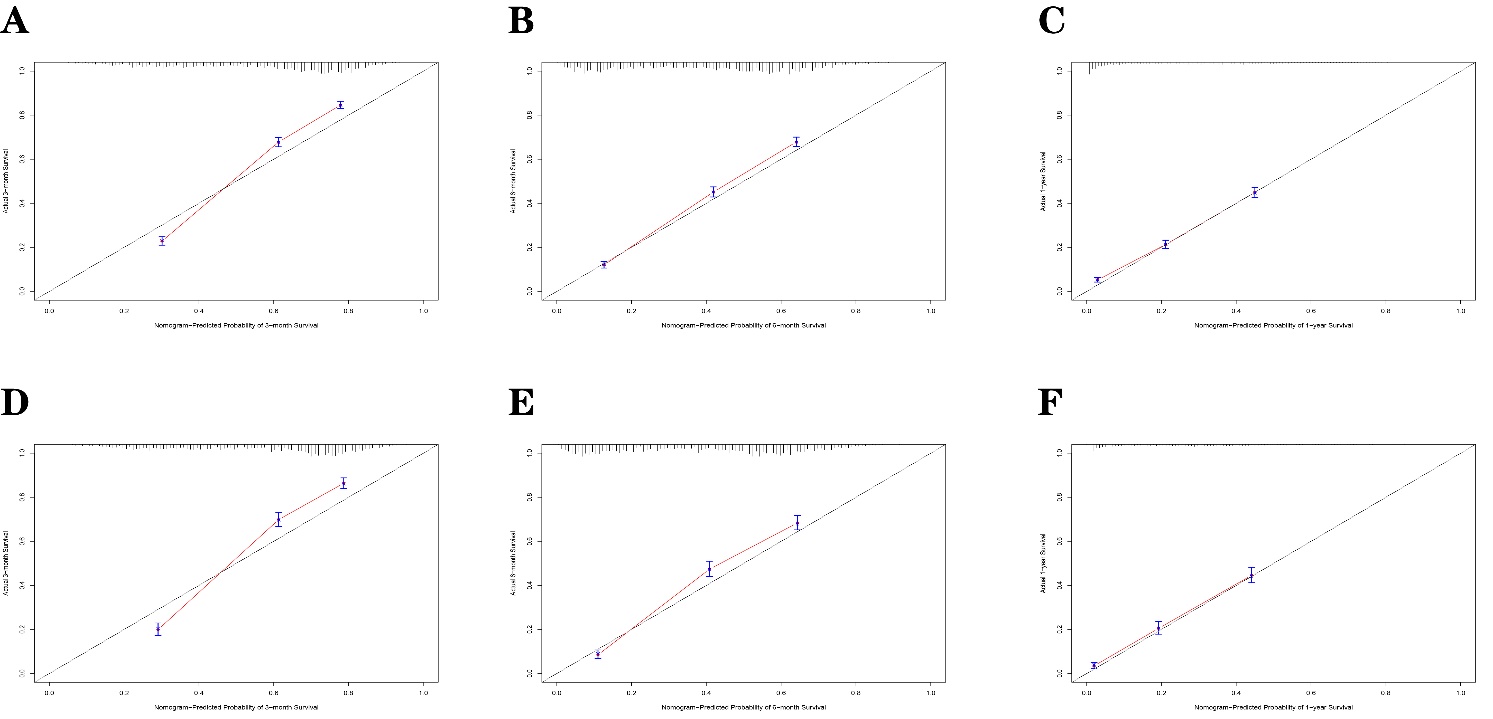


**Supplementary Figure 4** Decision curves. The decision curves analysis of the nomogram for 3-month, 6-month, and 1-year OS prediction of the training cohort (A-C), validation cohort (D-F).


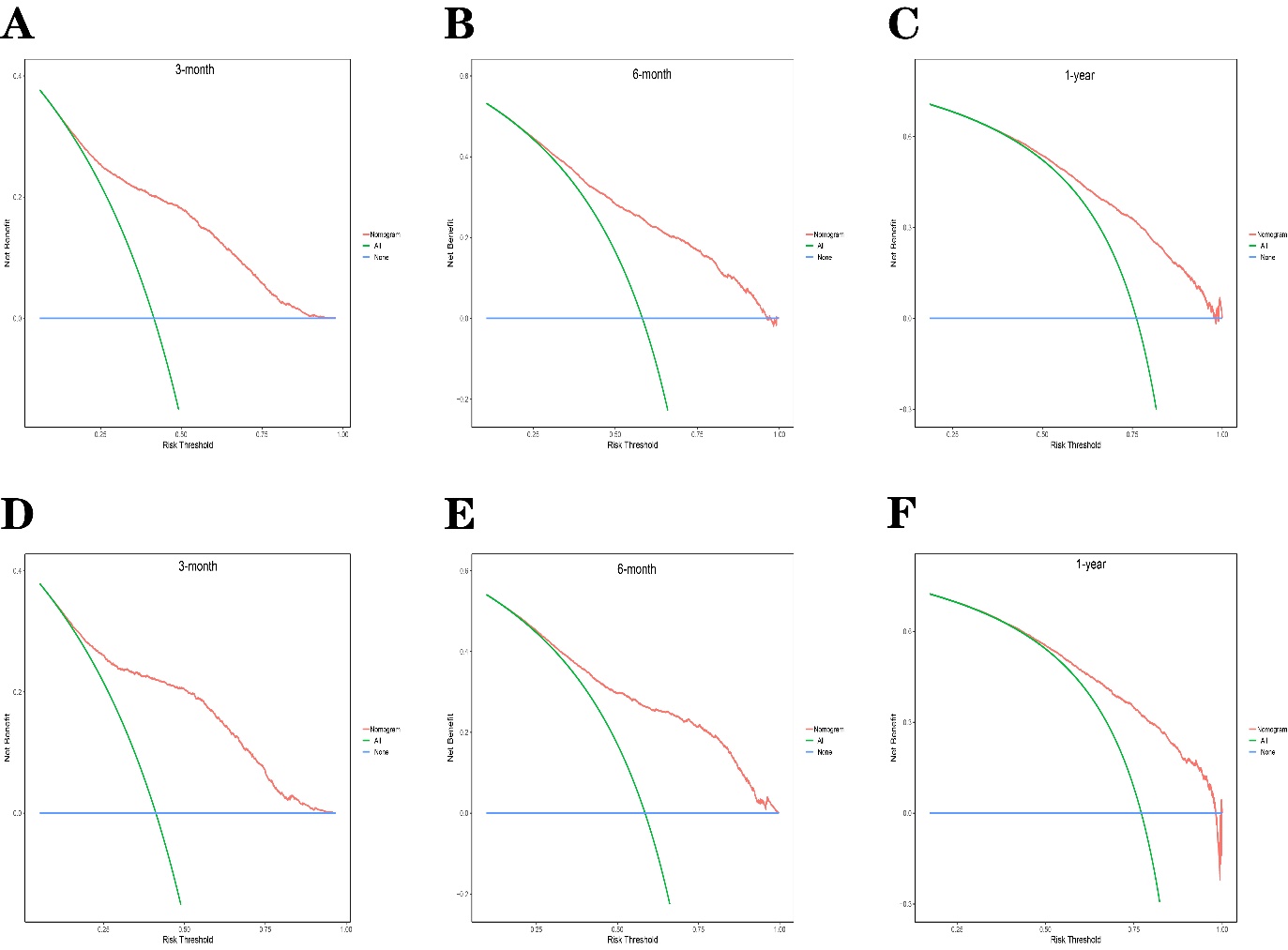


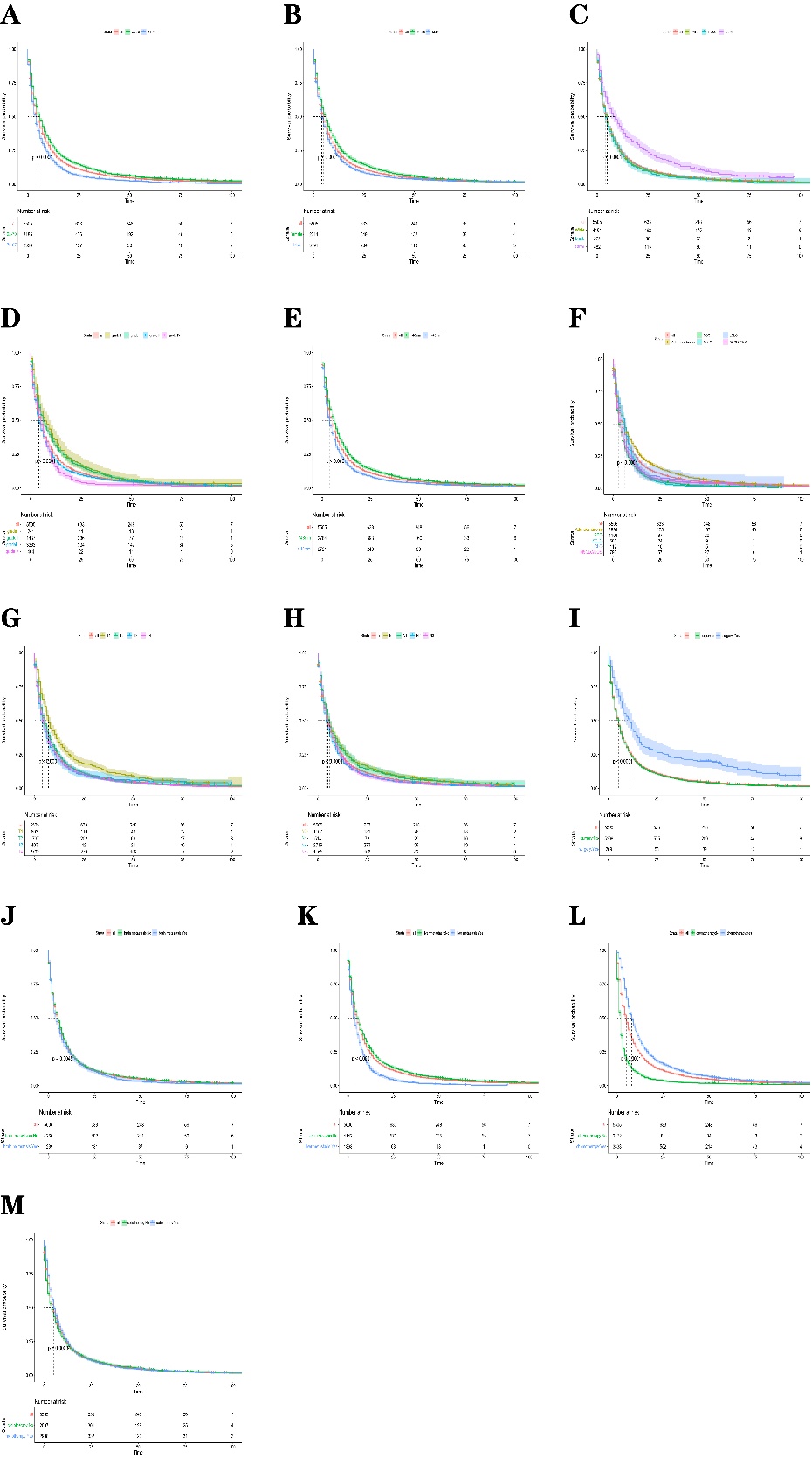
**Supplementary Figure 5** Predicted probability of OS by age (A), sex (B), race (C), grade (D), tumor size (E), histologic type (F), T stage (G), N stage (H), surgery (I), brain metastasis (J), liver metastasis (K), chemotherapy (L), radiotherapy (M) shown using Kaplan-Meier curves.
